# A systematic review of the incidence, risk factors and prognosis of acute exacerbation of systemic autoimmune disease-associated interstitial lung disease

**DOI:** 10.1186/s12890-021-01502-w

**Published:** 2021-05-05

**Authors:** Hiroyuki Kamiya, Ogee Mer Panlaqui

**Affiliations:** 1 Department of Respiratory Medicine, Tatebayashi Kosei Hospital, 262-1 Narushima-cho, Tatebayashi, Gunma 374-8533 Japan; 2 Department of Intensive Care Medicine, Northern Hospital, Melbourne, Australia

**Keywords:** Systemic autoimmune disease, Interstitial lung disease, Acute exacerbation, Risk, Prognosis, Review

## Abstract

**Introduction:**

Acute exacerbation (AE) is a devastating phenomenon and reported to be complicated with systemic autoimmune disease-associated interstitial lung disease (ILD). The aim of this study was to investigate the incidence and prognosis of AE of systemic autoimmune disease-ILD and clarify relevant clinical information predictive of these outcomes.

**Method:**

This study was designed as a systematic review and meta-analysis. A primary study except for a case report, which reported the incidence and/or prognosis of AE of systemic autoimmune disease-ILD, was eligible for the review. Electronic databases such as Medline and EMBASE were searched from 2002 through 23 February 2020. Two reviewers independently selected eligible reports and extracted relevant data. Risk of bias of individual studies was assessed similarly. The incidence and prognosis of the disease were analysed qualitatively. Univariate results of risk and prognostic factors were combined if feasible.

**Results:**

Out of a total of 2662 records, 24 studies were eligible. A total of 420 subjects with 45.7% of men developed AE of systemic autoimmune disease-ILD and the two major underlying systemic autoimmune diseases were rheumatoid arthritis (34.2%) and polymyositis/dermatomyositis (31.9%). The frequency ranged from 4.3 to 32.9% with the incident rate being 3.19 and 5.77 per 100 patient-years and all-cause mortality was between 30.0 and 58.3% at 90 days. Age at initial presentation was significantly associated with the development of AE of systemic autoimmune disease-ILD with an HR of 1.22 (95%CI 1.05–1.50) while a percentage of predicted diffusing capacity of the lung for carbon monoxide (%DLCO) was also significantly associated with the development of the disease with an HR of 0.95 (95%CI 0.90–1.00) and an OR of 0.97 (95%CI 0.95–0.99). Partial pressure of arterial oxygen/fraction of inspired oxygen ratio (PaO_2_/FiO_2_) at AE was significantly associated with all-cause mortality of AE of systemic autoimmune disease-ILD with an HR of 0.99 (95%CI 0.98–0.99).

**Conclusion:**

AE of systemic autoimmune disease-ILD was not uncommon and demonstrated dismal prognosis. Age at initial presentation and %DLCO were deemed as risk factors while PaO_2_/FiO_2_ at AE was considered as a prognostic factor of the disease.

*Registration* CRD42019138941.

**Supplementary Information:**

The online version contains supplementary material available at 10.1186/s12890-021-01502-w.

## Background

Interstitial lung disease (ILD) is a heterogeneous clinical entity, which is represented by interstitial pneumonia (IP) and pathologically defined as fibrosis mixed with varying degrees of inflammation in the interstitium of pulmonary parenchyma [1]. IP is classified as either idiopathic IPs (IIPs) with unknown aetiology or IPs secondary to other medical conditions such as systemic autoimmune disease, drug toxicity and dust exposure [2–4]. Systemic autoimmune disease-associated ILD is the most common among IPs with known causes [5]. Systemic autoimmune disease-ILD is usually manifested as a chronic disease that develops simultaneously or following the diagnosis of defined systemic autoimmune diseases [6] although ILD anticipates the onset of systemic autoimmune diseases in a non-negligible percentage of cases [7]. Although a complication of ILD generally worsens clinical course of systemic autoimmune diseases [8], the prognosis of systemic autoimmune disease-ILD is noted to be better than IIPs under the comparison of these two diseases with the same pathological patterns [9]. However, some studies reported acute exacerbation (AE) of systemic autoimmune disease-ILD, which demonstrated a devastating disease course [10]. AE of IP was first reported in patients with idiopathic pulmonary fibrosis (IPF) (pathologically usual interstitial pneumonia (UIP)) [11] although it was subsequently recognised that AE can develop in IPs with other pathological patterns such as non-specific interstitial pneumonia (NSIP) [12], which is the most common type for systemic autoimmune disease-ILD [13]. AE is characterised by an accelerated progression beyond its usually anticipated disease course [14] and responsible for approximately 40% of deaths of IPF [15]. However, clinical features of AE of systemic autoimmune disease-ILD have yet to be fully understood although its prognosis may be as grim as that of AE of IPF [16]. For example, it is unknown whether there is any predilection in the underlying IP patterns that are closely associated with the incidence of AE of systemic autoimmune disease-ILD. Whether the incidence of AE of systemic autoimmune disease-ILD varies depending on underlying systemic autoimmune diseases is also unclear. Furthermore, systemic autoimmune disease-ILD is usually treated by corticosteroid and/or immunosuppressive agents based on the underlying immunological abnormalities [17]. However, these prior treatments may get patients with systemic autoimmune disease-ILD to become more susceptible to AE under enhanced immunosuppression or help prevent the development of this intractable condition. As a result, there may be clinical difference between AE of systemic autoimmune disease-ILD and IPF. Therefore, the aim of this systematic review was to clarify current evidence regarding the incidence, risk factors and prognosis of AE of systemic autoimmune disease-ILD. The protocol of this study was registered with PROSPERO (International Prospective Register of Systematic Reviews) (CRD42019138941).

## Methods

This review was conducted and reported according to the Preferred Reporting Items for Systematic Reviews and Meta-Analyses (PRISMA) [18] and the Meta-analysis of Observational Studies in Epidemiology (MOOSE) statement [19].

### Eligibility criteria

Patients with AE of systemic autoimmune disease-ILD were eligible for this review. Systemic autoimmune diseases of interest consisted of rheumatoid arthritis (RA), systemic sclerosis (SSc), polymyositis/dermatomyositis (PM/DM) including clinically amyopathic DM (CADM), Sjögren’s syndrome (SS), systemic lupus erythematosus (SLE) and mixed connective tissue disease (MCTD), which were all diagnosed based on widely accepted classification criteria such as the 2010 American College of Rheumatology/European League Against Rheumatism criteria for RA [20]. Anti-neutrophil cytoplasmic antibody (ANCA)-associated vasculitides such as microscopic polyangiitis (MPA) were also eligible as a systemic autoimmune disease, which was diagnosed based on a previous consensus statement [21]. ILD was diagnosed radiologically and/or pathologically and its patterns were classified following the international classification criteria such as an official American Thoracic Society/European Respiratory Society statement [22]. Systemic autoimmune disease-ILD was defined as chronic IP that preceded or followed the diagnosis of defined systemic autoimmune diseases. As there were no established diagnostic criteria for AE of systemic autoimmune disease-ILD, the previous international group report for AE of IPF was applied to diagnose AE of systemic autoimmune disease-ILD, which consisted of acute worsening or development of dyspnoea (typically occurring within less than one month) and newly emerging bilateral ground glass opacity (GGO) and/or consolidation superimposed on background reticular or honeycomb patterns on high resolution computed tomography (HRCT) scans [23]. Although it was necessary to rule out cardiac failure or fluid overload as a cause of deterioration of systemic autoimmune disease-ILD, infections or other potential triggers did not need to be excluded, which accounted for both triggered and idiopathic cases [23]. Although the presence of underlying radiological and/or pathological changes consistent with chronic IP was required, they were not limited to UIP given the finding that systemic autoimmune disease-ILD is morphologically diverse [24]. Acute progressive form of IP at the first presentation was excluded if underlying chronic ILD complicated with systemic autoimmune diseases was not identified. In addition, PM/DM/CADM with acute worsening of ILD within 3 months after the diagnosis of the disease were also ineligible to exclude rapidly progressive ILD concomitant with the disease. ILD accompanied by undefined systemic autoimmune diseases or interstitial pneumonia with autoimmune features (IPAF) was not excluded unless it constituted the majority of subjects [25]. In cases where patients had multiple episodes of AE, only the first presentation of the disease was considered for further analysis.

Any clinical information, such as demographic features, symptoms, pulmonary functions, radiological findings and laboratory tests, was considered as potential risk and/or prognostic factors of AE of systemic autoimmune disease-ILD if they were investigated regarding their association with the incidence and/or prognosis of the disease in at least three studies. Prior treatment before the development of AE was also considered as risk and/or prognostic factors of AE of systemic autoimmune disease-ILD.

The primary outcomes of interest were the incidence and prognosis of AE of systemic autoimmune disease-ILD. The prognosis of AE of systemic autoimmune disease-ILD was defined as all-cause and pulmonary-cause mortality during a short period of time, which was determined in hospital and at 30 days (or 1 month). Long-term all-cause mortality, determined at 90 days (or 3 months), 6 months and 1 year after the diagnosis of the disease, was also evaluated as prognosis of AE of systemic autoimmune disease-ILD.

Primary studies of any type (excluding a case report) were eligible for the review if the incidence and/or prognosis of AE of systemic autoimmune disease-ILD was reported and quantitative data was available. Editorials, letters, review articles and conference proceedings were excluded. Research papers prior to 2002 were not considered because that year marked the time when the current classification system for IIPs, which is usually applied to classify systemic autoimmune disease-ILDs, was first reported [26]. Only articles published in English were reviewed.

### Search strategy

Electronic databases, i.e., Medline and EMBASE, were searched by the reviewers (H.K. and O.M.P.) using subject headings and text words related to study population such as ‘interstitial pneumonia’, ‘connective tissue disease’ and ‘acute exacerbation’ (e-Appendix). The search process was guided by a review in a similar research area identified in the Cochrane Database of Systematic Reviews (CDSR). Methodology filters were not used to avoid limiting the sensitivity of the search. The Science Citation Index Expanded was also consulted using terms adapted from the previous search of Medline and EMBASE. The search period covered 2002 through to the 1^st^ of August 2019 and was extended up to the 23rd of February 2020. The reference lists of eligible studies were also hand-searched to consolidate the implemented search strategy. Grey literature in this subject area was identified using Google Scholar [27].

### Study selection and data collection process

Two reviewers (H.K. and O.M.P.) independently examined titles and abstracts of all retrieved articles to identify eligible reports. The same reviewers also extracted relevant data based on the modified data extraction form, which was previously published in a protocol paper for a systematic review. [28] Any uncertainty or disagreement between the reviewers arising from these processes was resolved by discussion. The following data was extracted from each eligible study: first author’s name, year of publication, study location, study design, sample size and its demographic features, outcomes of interest, absolute numbers of outcome, risk and prognostic factors, methods of statistical analysis, summary statistics and items associated with a risk of bias.

### Risk of bias in individual studies

The Quality in Prognostic Studies (QUIPS) tool was modified and applied to assess a risk of bias in individual studies [29] since prognostic studies broadly include research predicting both the occurrence of a certain disease or condition (risk) and the outcome of the disease (prognosis) [30]. The QUIPS consists of six domains, one of which corresponds to ‘prognostic factor measurement’. As this review focused on both the incidence and prognosis of AE of systemic autoimmune disease-ILD, this domain was modified as ‘risk factor or prognostic factor measurement’ to address both of these outcomes. The other domains were adopted without any modification as they were applicable to studies of both risk and prognosis. Each domain received an individual bias rating (low, moderate or high), with an overall risk of bias determined based on the combined rating of all domains. For example, a study showing a low risk of bias across all domains was deemed as a low risk of bias overall [29].

### Statistical analysis

#### Summary statistics

The effect of risk and prognostic factors was summarised using either hazard ratios (HRs) derived from cox proportional hazards models [31] or odds ratios (ORs) derived from logistic regression models [32]. If an outcome was presented only using a log-rank test based on Kaplan–Meier survival curves, the HRs were to be calculated, as previously reported [33]. If the results were presented using both statistical models and log-rank tests, the former results were prioritised. The ORs or the risk ratios (RRs) were calculated manually based on absolute numbers of the outcome of interest across the two comparative groups if the effect of risk and/or prognostic factors was not directly available. Where risk and/or prognostic factors were handled as continuous variables, their effect was presented using either relative values or mean differences (MDs) between the two comparative groups.

#### Data synthesis

The effect estimates of risk and prognostic factors were statistically synthesised separately if it was presented using the same statistics in three or more studies. The results were summarised separately using a relative value, i.e., HRs, ORs or RRs or an absolute value, i.e., MDs. When the median, range or interquartile range was presented for continuous variables, they were converted to the mean with standard deviation, using a formula reported by a previous study [34]. Only unadjusted effect estimates for risk and prognostic factors were combined. The results of multivariate analysis were described qualitatively because adjusted factors were diverse between studies and pooling these data could be misleading. If meta-analysis was feasible from the collated data, it was conducted using a random-effects model employing the DerSimonian and Laird method [35]. Meta-analysis was conducted using the statistical software package, Review Manager (RevMan) Version 5.3 (Copenhagen: The Nordic Cochrane Centre, The Cochrane Collaboration, 2014). Statistical significance was considered with a *p* value of < 0.05. If combining data was deemed inappropriate due to a small number of studies with the same summary statistics, the results were reported qualitatively.

#### Heterogeneity between studies

Between-study variance was estimated using τ^2^ and assessed using both Q statistics and I^2^. Statistical significance for heterogeneity was considered with a *p* value of < 0.1 due to the low power of the test. Magnitude of heterogeneity was categorised as low (≤ 30%), moderate (> 30 ≤ 50%), considerable (> 50 ≤ 70%) and substantial (> 70%) [36]. The 95% prediction interval (PI) was presented alongside with the 95% confidence interval (CI) if combined results were presented and statistically significant heterogeneity was identified between studies [37]. To better interpret the sources of heterogeneity, subgroup analysis was to be conducted based on radiological and/or pathological patterns of IP (UIP or non-UIP), diagnosis of underlying systemic autoimmune diseases or study location (Asia or non-Asia). Sensitivity analysis was also considered focusing on studies with a low risk of bias alone.

#### Meta-biases

Small study bias (such as publication bias) was to be examined using graphical asymmetry of a funnel plot and the Egger’s test using Stata 14 (STATA Corp LLC., College Station, TX, USA) if 10 or more studies were available for meta-analysis [38]. Statistical significance was considered with a *p* value of < 0.1 due to the low power of the test. If publication bias was suspected, an adjusted summary effect was to be estimated using the trim and fill method [39].

### Confirmation of risk and prognostic factors

Risk and prognostic factors were confirmed if their effect estimates were in the same direction and statistically significant in the majority of studies by a multivariate analysis.

### Confidence in cumulative evidence

The credibility of evidence generated from this review was assessed by the Grades of Recommendation, Assessment, Development and Evaluation (GRADE) for prognosis [40]. The GRADE system was applied to both univariate and multivariate results of the final list of confirmed risk and prognostic factors since risk assessment was broadly considered as prognostic studies [30].

## Results

### Selection of studies

**Fig. 1:**
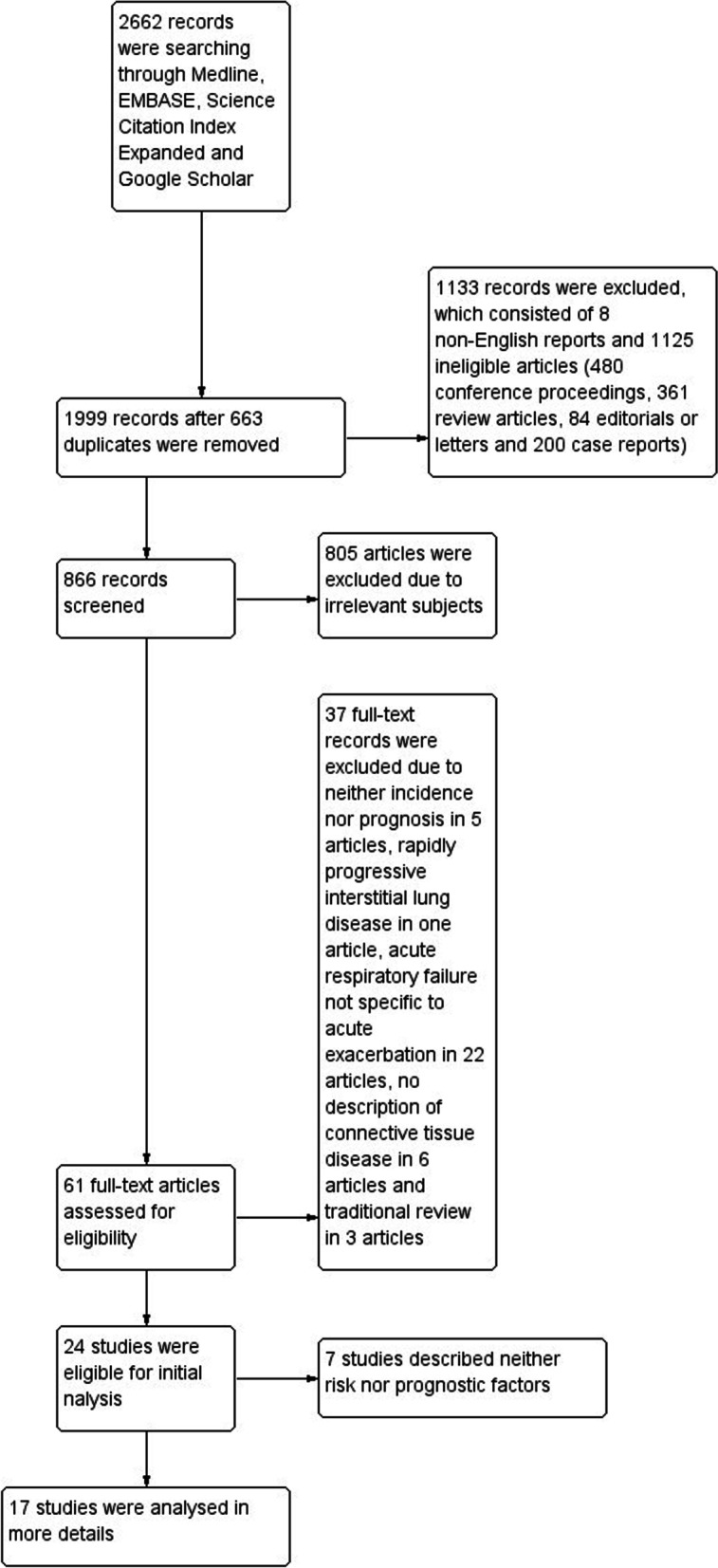
Study flow diagram. A total of 2662 reports were identified through Medline, EMBASE, Science Citation Index Expanded and Google Scholar. After excluding 663 duplicates, 8 non-English records, 1125 reports of ineligible types (consisting of 480 conference proceedings, 361 review articles, 84 editorials or letters and 200 case reports) and 805 irrelevant articles, the remaining 61 reports were obtained as full-texts. Out of these, 37 reports were excluded due to neither risk nor prognosis in 5 studies, rapidly progressive interstitial lung disease in one article, acute respiratory failure not specified as acute exacerbation in 22 studies, no description of systemic autoimmune disease in 6 studies and traditional reviews in 3 studies. Finally, 24 studies were eligible for this review. Of these, seven studies were excluded from risk of bias assessment and the analysis of risk or prognostic factors because no such information was available. The remaining 17 studies were analysed in more details to elucidate risk and/or prognostic factors of acute exacerbation of systemic autoimmune disease-associated interstitial lung disease

Out of a total of 2662 records identified through a search of four electronic databases, 61 records were retrieved as full-texts after excluding 663 duplicates, 8 non-English reports, 1125 reports of ineligible types and 805 irrelevant articles, and finally 24 studies were eligible for this review [41–64]. Of these, seven studies were excluded from risk of bias assessment and the analysis of risk or prognostic factors because no such information was available [41–47] (Fig. 1).

### Demographic features of eligible studies

Although three studies were conducted by one research group [50, 51, 62] and other two studies were reported by another team [43, 44], all of these reports were considered as a separate study due to a different target disease or a different period of enrolment. The largest number of included studies was conducted in Japan (n = 14), which was followed by South Korea and China (n = 3 for each) and the U.S.A, Canada, Italy and India (n = 1 for each) (Table 1). 22 studies were designed retrospectively, which was composed of retrospective cohort study (n = 17) and case–control study (n = 5) whereas the remaining two studies were of prospective cohort design. A total of 420 subjects with 45.7% of men developed AE of systemic autoimmune disease-ILD. The most prevalent underlying systemic autoimmune disease was RA (n = 123, 34.2%), which was followed by PM/DM (n = 115, 31.9% including 16 cases of CADM), SSc (n = 41, 11.4%), SS (n = 35, 9.7%), MPA or ANCA-associated vasculitis (n = 15, 4.2%) and MCTD (n = 12, 3.3%). The mean or median age at the onset of AE was between 45.8 and 74.5 years. The proportion of smoking history reported in 14 studies was distributed between 11.1 and 86.7% while that of radiological and/or pathological UIP pattern reported in 17 studies was between 13.3 and 100%. The frequency of AE of systemic autoimmune disease-ILD was reported in a total of 17 studies, which ranged from 4.3 to 32.9% and the incident rate was 3.19 and 5.77 per 100 patient-years in two studies [45, 55]. A total of 19 studies reported the prognosis, which included in-hospital all-cause mortality of 33.3–83.3% in three studies, 30-day all-cause mortality of 10.0–33.3% in two studies, 90-day all-cause mortality of 30.0–58.3% in five studies, 1-year all-cause mortality of 61.5% in one study and overall all-cause mortality of 16.7–100% in 12 studies (Table 1).

**Table 1:** Characteristics of included studies

Study	Country	Study design	Subjects (n) (M/F)	Diagnosis of systemic autoimmune disease (n)	Age (years) (at the onset of AE)	Smoking (n (%))	UIP pattern (n (%))^a^	Follow-up lengths (months)	Frequency of AE (n (%))/Incidence	All-cause mortality (n (%))
Isobe [41]	Japan	Case–control	2 (2/0)	RA2	74.0 ± 7.1	–	2 (100.0)	–	2/8 (25.0) among RA-ILD	2/2 (100.0) (overall)
Lim [42]	Korea	Retrospective-cohort	25	Not specified	–	–	–	–	25/76 (32.9)	–
Park [43]	Korea	Retrospective-cohort	4 (3/1)	RA3, SSc1	median 58 (range 47–68)	2 (50.0)	3 (75.0)	–	4/93 (4.3) among systemic autoimmune disease-ILD^g^	4/4 (100.0) (overall)
Song [44]	Korea	Retrospective-cohort	14 (8/6)	RA14	68.1 ± 7.8^d^	7 (50.0)	14 (100.0)	–	14/84 (16.7) among RA-ILD	13/14 (92.9) (overall)
Suzuki [45]	Japan	Retrospective-cohort	27	RA7, SSc11, PM/DM4, SS2, SLE2, MCTD1	–	–	–	–	27/205 (13.2) among systemic autoimmune disease-ILD^c^/3.19 per 100 patient-years,	–
Tachikawa [46]	Japan	Retrospective-cohort	15 (7/8)	RA6, SSc3, DM3, CADM3	63.3 ± 6.8	8 (57.1) (n = 14)	4 (26.7)	–	–	(33.0) (90 days)
Tomiyama [47]	Japan	Retrospective-cohort	13 (3/10)	SSc13	49.3 ± 12.7 (at the onset of SSc)	–	–	–	13/55 (23.6) among SSc-ILD	6/13 (46.2) (overall)
Akiyama [48]	Japan	Retrospective-cohort	6 (2/4)	RA6	66.8 ± 6.2 (at the start of treatment)	2 (40.0) (n = 5)	3 (50.0)	–	6/78 (7.7) among RA-ILD	–
Cao [49]	China	Retrospective-cohort	70 (35/35)	RA16, PM/DM8, SS25, MCTD6, ANCA-vasculitis5, IPAF10	65.8 ± 9.3^d^	18 (25.7)	70 (100.0)	–	70/1168 (6.0) among systemic autoimmune disease-ILD^g^	–
Enomoto [50]	Japan	Retrospective-cohort	15 (11/4)	RA9, SSc2, MPA3, MPA + SS1	median 71 (range 57–85)	13 (86.7)	–	Median 56 (range 0–228)^e^	–	5/15 (33.3) (1 month)
										7/15 (46.7) (3 months)
										11/15 (73.3) (overall)
Hozumi [51]	Japan	Retrospective-cohort	11 (6/5)	RA11	median 72 (range 60–86)	9 (81.8)	6 (54.5)	Median 8.5 (range 1–17) (years)^f^	11/51 (21.6) among RA-ILD	7/11 (63.6) (overall)
Ichiyasu [52]	Japan	Retrospective-cohort	38	RA6, SSc1, PM5, DM17, SS1, SLE1, MCTD1, MPA6	–	–	–	–	–	(58.3) (90 days)
Ishikawa [53]	Japan	Case–control	9 (3/6)	Not specified	56.4 ± 15.6	–	–	–	–	4/9 (44.4) (3 months)
Liang [54]	China	Case–control	64 (25/39)	PM15, DM36, CADM13	57.7 ± 11.9^d^	14 (21.9)	15 (23.4)	–	64/483 (13.3) among IIM-ILD	25/64 (39.1) (in-hospital or within 2 weeks after discharge)
Manfredi [55]	Italy	Prospective-cohort	9 (3/6)	RA1, SSc1, SS1, MCTD1, SSc + DM1, RA + SS1, ANCA-vasculitis1, IPAF2	67.8 ± 8.5 (at the diagnosis of ILD)	1 (11.1)	6 (66.7)	23.9 ± 10.9^f^	9/78 (11.5) among systemic autoimmune disease-ILD^g^/5.77 per 100 patient-years	5/9 (55.6) (overall)
Okamoto [56]	Japan	Retrospective-cohort	4	SSc4	–		4 (100.0)	–	4/33 (12.1) among SSc-ILD	4/4 (100.0) (overall)
Ota [57]	Japan	Retrospective-cohort	12 (4/8)	RA12	median 74.5 (range 50–80)	4 (33.3)	10 (83.3)	Median 19.5 (range 9–88)^e^	–	2/12 (16.7) (overall)
Parambil [58]	USA	Case–control	6 (2/4)	RA4, PM2	medina 68 (range 43–76)	3 (50.0)	2 (33.3)	–	–	5/6 (83.3) (in-hospital)
Silva [59]	Canada	Case–control	8 (2/6)	RA3, PM/DM4, SS1	median 62 (range 53–72)	–	5 (62.5)	–	–	6/8 (75.0) (overall)
Singh [60]	India	Prospective-cohort	15 (5/10)	RA1, SSc5, DM3, SS3, MCTD3	45.8 ± 13.9	4 (26.7)	2 (13.3)	24 ± 18.1^f^	15/105 (14.3) among systemic autoimmune disease-ILD^b^	5/15 (33.3) (in-hospital)
Su [61]	China	Retrospective-cohort	26	Not specified	–	–	–	–	26/161 (16.1) among systemic autoimmune disease-ILD^g^	16/26 (61.5) (1 year)
Suda [62]	Japan	Retrospective-cohort	6 (4/2)	RA5, SS1	65.7 ± 5.3	5 (83.3)	3 (50.0)	Mean 6.0 (years)^f^	6/83 (7.2) among systemic autoimmune disease-ILD^g^	5/6 (83.3) (overall)
Toyoda [63]	Japan	Retrospective-cohort	10 (7/3)	RA6, PM/DM2, SLE1, SS1	median 73 (range 61–83)	6 (60.0)	8 (80.0)	–	10/155 (6.5) among systemic autoimmune disease-ILD^g^	1/10 (10.0) (30 days)
										3/10 (30.0) (90 days)
										7/10 (70.0) (overall)
Yamakawa[64]	Japan	Retrospective-cohort	11	RA11	–	–	3 (27.3)	–	11/96 (11.5) among RA-ILD	–

Values indicate mean ± SD or number (percentage) unless otherwise specified

AE, acute exacerbation; ANCA, anti-neutrophil cytoplasmic antibody; CADM, clinically amyopathic dermatomyositis; DM, dermatomyositis; IIM, idiopathic inflammatory myositis; ILD, interstitial lung disease; IPAF, interstitial pneumonia with autoimmune features; IQR, interquartile range; MCTD, mixed connective tissue disease; MPA, microscopic polyangiitis; PM/DM, polymyositis/dermatomyositis; RA, rheumatoid arthritis; SLE, systemic lupus erythematosus; SS, Sjögren’s syndrome; SSc, systemic sclerosis; UIP, usual interstitial pneumonia

^a^Indicating radiological and/or pathological UIP pattern

^b^SSc 5/30 (16.7%), MCTD 3/28 (10.7%), RA 1/16 (6.3%), DM 3/9 (33.3%), SS 3/6 (50.0%), others 0/16 (0.0%)

^c^SSc 11/42 (26.2%), RA 7/71 (9.9%), PM/DM 4/42 (9.5%), SLE 2/5 (40.0%), SS 2/24 (8.3%), MCTD 1/6 (16.7%), others 0/15 (0.0%)

^d^Unknown point in time

^e^After the development of acute exacerbation

^f^Before the development of acute exacerbation

^g^Unclear underlying systemic autoimmune disease

### Risk of bias in individual studies

‘Study confounding’ and ‘statistical analysis and reporting’ were rated as high risk of bias in the majority of included studies due to no or insufficient explanation of confounders and lack of information of multivariate models. As a result, all studies were considered as being subject to methodological limitations (Table 2).

**Table 2:** Risk of bias in individual studies

Study	Study participation	Study attrition	Risk factor measurement^a^	Outcome measurement	Study confounding	Statistical analysis and reporting
Akiyama [48]	Low risk	**High risk**	Moderate risk	Low risk	**High risk**	**High risk**
Cao [49]	Low risk	**High risk**	Moderate risk	Low risk	Moderate risk	Moderate risk
Hozumi [51]	Low risk	**High risk**	Moderate risk	Low risk	Moderate risk	Moderate risk
Ishikawa [53]	Moderate risk	Low risk	Low risk	Low risk	**High risk**	**High risk**
Liang [54]	Low risk	Low risk	**High risk**	Low risk	**High risk**	Low risk
Manfredi [55]	Low risk	**High risk**	Moderate risk	Low risk	**High risk**	**High risk**
Okamoto [56]	**High risk**	Low risk	**High risk**	Low risk	**High risk**	**High risk**
Silva [59]	Moderate risk	Low risk	Low risk	Low risk	**High risk**	**High risk**
Singh [60]	Low risk	Low risk	Low risk	Low risk	Moderate risk	**High risk**
Su [61]	Moderate risk	Low risk	Low risk	Low risk	**High risk**	**High risk**
Suda [62]	Low risk	**High risk**	Moderate risk	Low risk	**High risk**	**High risk**
Yamakawa [64]	Moderate risk	**High risk**	Low risk	Low risk	Moderate risk	**High risk**
**Study**	**Study participation**	**Study attrition**	**Prognostic factor measurement**^**a**^	**Outcome measurement**	**Study confounding**	**Statistical analysis and reporting**
Cao [49]	Low risk	**High risk**	Moderate risk	Low risk	Moderate risk	Moderate risk
Enomoto [50]	Moderate risk	**High risk**	Moderate risk	Low risk	**High risk**	Moderate risk
Ichiyasu [52]	**High risk**	**High risk**	Low risk	Low risk	**High risk**	**High risk**
Liang [54]	Low risk	Low risk	Moderate risk	Low risk	Moderate risk	Low risk
Manfredi [55]	Low risk	**High risk**	Moderate risk	Low risk	**High risk**	**High risk**
Ota [57]	Low risk	Low risk	Moderate risk	Low risk	Moderate risk	**High risk**
Parambil [58]	Low risk	Low risk	**High risk**	Low risk	**High risk**	**High risk**
Silva [59]	Moderate risk	Low risk	Low risk	Low risk	**High risk**	**High risk**
Singh [60]	Low risk	Low risk	Low risk	Low risk	Moderate risk	**High risk**
Toyoda [63]	Low risk	**High risk**	**High risk**	Low risk	**High risk**	**High risk**

Bold in text indicating a high risk of bias

^a^Separately presented depending on whether a risk or prognostic factor was reported

### Risk factors of AE of systemic autoimmune disease-ILD

**Fig. 2:**
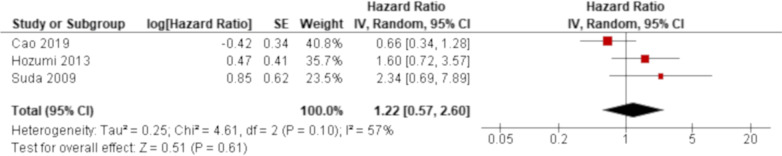
Forrest plot of the result of univariate analysis for smoking history (ever-smoking) as a risk factor. The result of univariate analysis in three studies was pooled for meta-analysis. Smoking history (ever-smoking) was not significantly associated with the development of acute exacerbation (AE) of systemic autoimmune disease-associated interstitial lung disease (ILD) with a hazard ratio (HR) of 1.22 (95% confidence interval: 0.57–2.60, *p* = 0.61/95% prediction interval: 0.0004–3734.1). There was considerable heterogeneity (chi^2^ = 4.61, *p* = 0.10, I^2^ = 57%). Although both Cao 2019 and Suda 2009 enrolled systemic autoimmune disease-ILDs, the former study demonstrated that Sjögren’s syndrome was the largest in number as the underlying disease of AE whereas it was rheumatoid arthritis (RA) in the latter study. Hozumi 2013 only enrolled RA-ILD cases

**Fig. 3:**
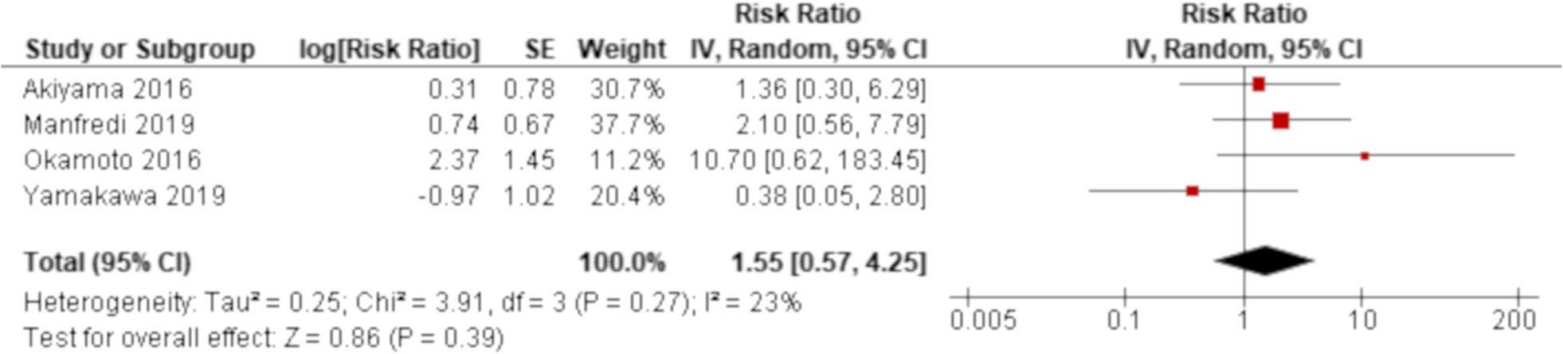
Forrest plot of the result of univariate analysis for radiological usual interstitial pneumonia (UIP) pattern as a risk factor. The result of univariate analysis in four studies was pooled for meta-analysis. Radiological UIP pattern was not significantly associated with the development of acute exacerbation (AE) of systemic autoimmune disease-associated interstitial lung disease (ILD) with a risk ratio (RR) of 1.55 (95% confidence interval: 0.57–4.25, *p* = 0.39). There was mild and statistically non-significant heterogeneity (chi^2^ = 3.91, *p* = 0.27, I^2^ = 23%)

A total of 8 clinical information was reported in at least three studies and selected as a potential risk factor for the development of AE of systemic autoimmune disease-ILD. These were age, sex (men), smoking history, forced vital capacity (FVC), diffusing capacity of the lung for carbon monoxide (DLCO), radiological UIP pattern (on HRCT), and corticosteroid and immunosuppressive therapy before AE. All of these factors were reported by a univariate analysis (Table 3). Conversely, pulmonary hypertension, the extent of ILD, the onset of ILD (ILD preceding or not) and a prior treatment using biologics or anti-fibrotic agents were not selected as potential risk factors because only less than three studies reported the risk of AE related to that clinical information (e-Table). Although a combined analysis of univariate results was conducted for smoking history and radiological UIP pattern, neither of them demonstrated significant results (Figs. 2, 3, respectively). None of the potential risk factors were significantly associated with the development of AE of systemic autoimmune disease-ILD in studies excluded from meta-analysis aside from age in two studies with HRs of 1.11 (95%CI 1.01–1.20) [51] and 1.19 (95%CI 1.04–1.36) [62] and DLCO in two studies with MDs of − 8.70 (95%CI − 14.4 to − 3.01) [54] and − 12.3 (95%CI − 24.3 to − 0.32) [55] and radiological UIP pattern and corticosteroid therapy before AE in one study each with an HR of 1.95 (95%CI 1.07–3.63) [51] and an HR of 0.42 (95%CI 0.22–0.80) [49], respectively (Table 3).

**Table 3:** Risk factors of acute exacerbation of systemic autoimmune disease-associated interstitial lung disease by univariate analysis

Potential risk factors^a^	Studies^b^	Effect estimates (95% confidence interval)^c^
*Demographic features*		
Age	Hozumi [51]	**HR 1.11 (1.01–1.20) (year) (at ILD diagnosis)**
	Suda [62]	**HR 1.19 (1.04–1.36) (year) (at initial presentation)**
	Cao [49]	HR 1.01 (0.97–1.04) (year)^d^
	Akiyama [48]	MD − 2.60 (− 9.17 to 3.97) (year) (at the start of ILD treatment)
	Manfredi [55]	MD 5.00 (− 3.06 to 13.1) (year) (at ILD diagnosis)
	Liang [54]	MD 0.40 (− 3.48 to 4.28) (year)^d^
Sex (men)	Akiyama [48]	RR 1.36 (0.27–6.94)
	Manfredi [55]	RR 0.94 (0.25–3.50)
	Cao [49]	HR 0.75 (0.43–1.30)^e^
	Hozumi [51]	HR 0.90 (0.49–1.69)
	Suda [62]	HR 1.31 (0.53–3.25)
	Liang [54]	OR 1.00 (0.54–1.85)
Smoking history (ever-smoking vs. non-smoking)	Meta-analysis (n = 3)	HR 1.22 (0.57–2.60)
	Akiyama [48]	RR 1.19 (0.21–6.60)
	Manfredi [55]	RR 0.16 (0.02–1.23)
	Liang [54]	OR 1.10 (0.53–2.28)
*Pulmonary function (before acute exacerbation)*		
FVC	Cao [49]	HR 0.86 (0.56–1.31) (L)
	Hozumi [51]	HR 1.02 (0.99–1.06) (% of predicted value)
	Manfredi [55]	MD − 7.60 (− 23.0 to 7.81) (% of predicted value)
DLCO	Cao [49]	HR 1.00 (0.97–1.03) (% of predicted value)
	Suda [62]	HR 1.05 (0.98–1.21) (% of predicted value)
	Liang [54]	**MD **− **8.70 (**− **14.4 to 3.01) (% of predicted value)**
	Manfredi [55]	**MD **− **12.3 (**− **24.3 to 0.32) (% of predicted value)**
*Underlying radiological features*		
UIP pattern on HRCT	Meta-analysis (n = 4)	RR 1.55 (0.57–4.25)
	Hozumi [51]	**HR 1.95 (1.07–3.63)**
	Liang [54]	OR 1.40 (0.67–2.91)
*Pre-treatment*		
Corticosteroid	Cao [49]	**HR 0.42 (0.22–0.80)**
	Hozumi [51]	HR 0.97 (0.53–1.92)
	Akiyama [48]	RR 3.48 (0.43–28.4)
	Manfredi [55]	RR 2.41 (0.15–38.0)
	Liang [54]	OR 1.04 (0.54–2.01)
Immunosuppressive agents	Cao [49]	HR 0.73 (0.42–1.25)
	Hozumi [51]	HR 0.76 (0.35–1.41)
	Akiyama [48]	RR 0.45 (0.06–3.65)
	Manfredi [55]	RR 7.21 (0.44–118.6)

Bold in text indicating statistical significance

CRP, C-reactive protein; DLCO, diffusing capacity of the lung for carbon monoxide; FVC, forced vital capacity; HR, hazard ratio; HRCT, high resolution computed tomography; ILD, interstitial lung disease; KL-6, Krebs von den Lungen-6; MD, mean difference; OR, odds ratio; RR, risk ratio; UIP, usual interstitial pneumonia

^a^Any clinical information that was reported in at least three studies

^b^Where combined results were presented, it was designated as meta-analysis, otherwise each study was presented

^c^MD was calculated as a difference between subjects with and without acute exacerbation

^d^Unknown point in time

^e^A comparison is unknown

Only two out of 8 potential risk factors were reported by a multivariate analysis (Table 4). Age at initial presentation was significantly associated with the development of AE of systemic autoimmune disease-ILD with an HR of 1.22 (95%CI 1.05–1.50) in one study [62]. A percentage of predicted DLCO (%DLCO) was also significantly associated with the development of the disease in two studies with an HR of 0.95 (95%CI 0.90–1.00) [55] and an OR of 0.97 (95%CI 0.95–0.99) [54] (Table 4). Based on the result of these multivariate analyses, age at initial presentation and %DLCO were deemed as a risk factor of AE of systemic autoimmune disease-ILD.

**Table 4:** Risk and prognostic factors of acute exacerbation of systemic autoimmune disease-associated interstitial lung disease by multivariate analysis

Potential risk factors^a^	Study	Effect estimates (95% confidence interval)
**Age**	Suda [62]	**HR 1.22 (1.05–1.50) (year) (at initial presentation)**
**DLCO (before acute exacerbation)**	Manfredi [55]	**HR 0.95 (0.90–1.00) (% of predicted value)**
	Liang [54]	**OR 0.97 (0.95–0.99) (% of predicted value)**
**Potential prognostic factors**^**a**^	**Study**	**Effect estimates (95% confidence interval)**
**PaO**_**2**_**/FiO**_**2**_** (at acute exacerbation)**	Cao [49]	**HR 0.99 (0.98–0.99)**

Bold in text indicating statistical significance and risk or prognostic factors of acute exacerbation of systemic autoimmune disease-associated interstitial lung disease that were confirmed in this review

DLCO, diffusing capacity of the lung for carbon monoxide; HR, hazard ratio; OR, odds ratio; PaO_2_/FiO_2_, partial pressure of arterial oxygen/fraction of inspired oxygen ratio

^a^Any clinical information that was reported in at least three studies

### Prognostic factors of AE of systemic autoimmune disease-ILD

**Fig. 4:**
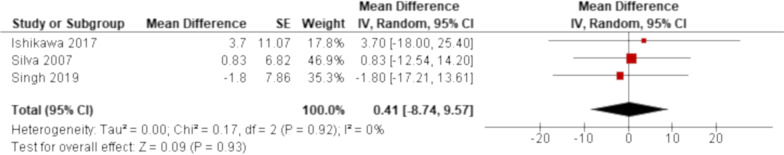
Forrest plot of the result of univariate analysis for age at the onset of acute exacerbation as a prognostic factor. The result of univariate analysis in three studies was pooled for meta-analysis. There was no significant difference in age at the onset of acute exacerbation (AE) between decedents and survivors of AE of systemic autoimmune disease-associated interstitial lung disease (ILD) with a mean difference (MD) of 0.41 (95% confidence interval: − 8.74 to 9.57, *p* = 0.93). There was no heterogeneity (chi^2^ = 0.17, *p* = 0.92, I^2^ = 0%)

**Fig. 5:**
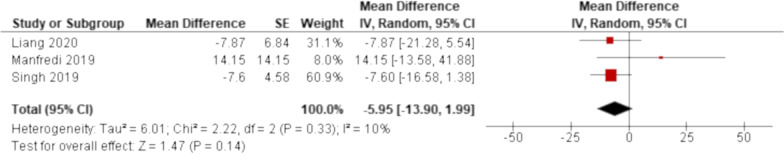
Forrest plot of the result of univariate analysis for a percentage of predicted forced vital capacity (%FVC) as a prognostic factor. The result of univariate analysis in three studies was pooled for meta-analysis. There was no significant difference in %FVC between decedents and survivors of acute exacerbation (AE) of systemic autoimmune disease-associated interstitial lung disease (ILD) with a mean difference (MD) of − 5.95 (95% confidence interval: − 13.90 to 1.99, *p* = 0.14). There was mild and statistically non-significant heterogeneity (chi^2^ = 2.22, *p* = 0.33, I^2^ = 10%)

**Fig. 6:**
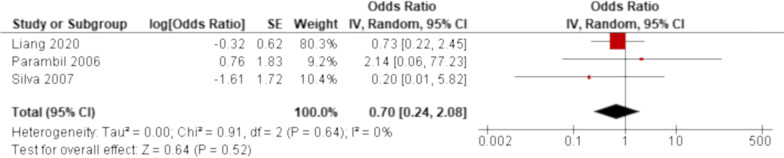
Forrest plot of the result of univariate analysis for radiological usual interstitial pneumonia (UIP) pattern as a prognostic factor. The result of univariate analysis in three studies was pooled for meta-analysis. Radiological UIP pattern was not significantly associated with all-cause mortality of acute exacerbation (AE) of systemic autoimmune disease-associated interstitial lung disease (ILD) with an odds ratio (OR) of 0.70 (95% confidence interval: 0.24 to 2.08, *p* = 0.52). There was no heterogeneity (chi^2^ = 0.91, *p* = 0.64, I^2^ = 0%)

A total of seven clinical information was reported in at least three studies and selected as a potential prognostic factor for all-cause mortality of AE of systemic autoimmune disease-ILD. These were age, sex, smoking history, FVC, radiological UIP pattern (on HRCT), partial pressure of arterial oxygen/fraction of inspired oxygen ratio (PaO_2_/FiO_2_) and lactate dehydrogenase (LDH). All of these factors were reported by a univariate analysis (Table 5). Conversely, white blood cell (WBC) count at the onset of AE was not selected as a potential prognostic factor because only less than three studies reported the prognosis of AE of systemic autoimmune disease-ILD related to this clinical information (e-Table). Although a combined analysis of univariate results was conducted for age, FVC and radiological UIP pattern, none of them demonstrated significant results (Figs. 4, 5, 6, respectively). None of the potential prognostic factors were significantly associated with all-cause mortality in studies excluded from meta-analysis aside from PaO_2_/FiO_2_ in one study with an HR of 0.989 (95%CI 0.985–0.994) [49] and LDH in the same study with an HR of 1.004 (95%CI 1.002–1.005) [49] (Table 5).

**Table 5:** Prognostic factors of acute exacerbation of systemic autoimmune disease-associated interstitial lung disease by univariate analysis

Potential prognostic factors^a^	Studies (n)^b^	Effect estimates (95% confidence interval)^c^
*Demographic features*		
Age (at acute exacerbation)	Meta-analysis (n = 3)	MD 0.41 (− 8.74 to 9.57) (year)
	Manfredi [55]	MD 6.55 (− 4.40 to 17.5) (year)^d^
	Liang [54]	MD 0.00 (− 6.49 to 6.49) (year) ^e^
	Enomoto [50]	HR 1.03 (*p* = 0.50) (year)
Sex (men)	Enomoto [50]	HR 3.19 (*p* = 0.29)
	Ishikawa [53]	OR 0.50 (0.03–8.95)
	Liang [54]	OR 1.85 (0.66–5.17)
	Singh [60]	RR 1.33 (0.32–5.58)
Smoking history (ever-smoking vs. non-smoking)	Singh [60]	RR 1.83 (0.46–7.25)
	Liang [54]	OR 1.22 (0.37–4.07)
	Parambil [58]	OR 4.20 (0.12–152.0)
*Pulmonary function (before acute exacerbation)*		
FVC	Meta-analysis (n = 3)	MD − 5.95 (− 13.9 to 1.99) (% of predicted value)
	Enomoto [50]	HR 1.07 (*p* = 0.35) (%of predicted value)
*Underlying radiological features*		
UIP pattern on HRCT	Meta-analysis (n = 3)	OR 0.70 (0.24–2.08)
	Manfredi [55]	RR 2.00 (0.37–10.9)
	Singh [60]	RR 0.42 (0.03–5.78)
*Laboratory findings (at acute exacerbation)*		
PaO_2_/FiO_2_	Cao [49]	**HR 0.989 (0.985–0.994)**
	Enomoto [50]	HR 0.99 (*p* = 0.18)
	Manfredi [55]	MD − 18.3 (− 77.4 to 40.9)
LDH	Cao [49]	**HR 1.004 (1.002–1.005) (U/L)**
	Enomoto [50]	HR 1.001 (*p* = 0.63) (IU/L)
	Liang [54]	MD 24.2 (− 86.2 to 134.5) (U/L)

Bold in text indicating statistical significance

FVC, forced vital capacity; HR, hazard ratio; HRCT, high resolution computed tomography; ILD, interstitial lung disease; LDH, lactate dehydrogenase; MD, mean difference; OR, odds ratio; PaO_2_/FiO_2_, partial pressure of arterial oxygen/fraction of inspired oxygen ratio; RR, risk ratio; UIP, usual interstitial pneumonia

^a^Any clinical information that was reported in at least three studies

^b^Where combined results were presented, it was designated as meta-analysis, otherwise each study was presented

^c^MD was calculated as a difference between decedents and survivors

^d^Age at ILD diagnosis

^e^Unknown point in time

Only one out of seven potential prognostic factors were reported by a multivariate analysis (Table 4). PaO_2_/FiO_2_ at the onset of AE was significantly associated with all-cause mortality of AE of systemic autoimmune disease-ILD with an HR of 0.99 (95%CI 0.98–0.99) in one study [49] (Table 4). Based on the result of this multivariate analysis, PaO_2_/FiO_2_ at the onset of AE was deemed as a prognostic factor of AE of systemic autoimmune disease-ILD.

### Additional analysis

Subgroup analysis was not undertaken due to the small number of included studies. Sensitivity analysis could not be conducted because no studies were deemed as low risk of bias. Small study bias such as publication bias could not be assessed because the designated minimum number of studies (≥ 10) was not available for any meta-analysis in this review.

### Quality of evidence

Due to the study limitation that was identified in all included studies and publication bias that was assumed to exist for prognostic studies [65], the GRADE system rated the quality of evidence for all of the identified risk and prognostic factors as either low or very low (Table 6).

**Table 6:** Assessment of quality of evidence of risk and prognostic factors by the Grades of Recommendation, Assessment, Development and Evaluation (GRADE) system

GRADE factors										
Risk factors^a^	Analysis^b^	Phase	Study limitations	Inconsistency^c^	Indirectness	Publication bias	Imprecision	Moderate/large effect size	Dose response gradient	Overall quality
*Outcome: all-cause mortality*										
Age	Uni	1	+	−	−	+	−	−	−	Low
	Multi	1	+	N/A	−	+	−	−	−	Very low
DLCO	Uni	1	+	+	−	+	−	+	−	Very Low
	Multi	1	+	−	−	+	−	−	−	Very low

GRADE factors										
Prognostic factors^a^	Analysis^b^	Phase	Study limitations	Inconsistency^c^	Indirectness	Publication bias	Imprecision	Moderate/large effect size	Dose response gradient	Overall quality
*Outcome: all-cause mortality*										
PaO_2_/FiO_2_	Uni	1	+	−	−	+	−	−	−	Very low
	Multi	1	+	N/A	−	+	−	−	−	Very low

DLCO, diffusing capacity of the lung for carbon monoxide; PaO_2_/FiO_2_, partial pressure of arterial oxygen/fraction of inspired oxygen ratio

^a^A total of two risk factors and one prognostic factor of acute exacerbation of systemic autoimmune disease-associated interstitial lung disease were identified based on consistent and statistically significant results in multivariate analyses among clinical information that were reported by at least three studies

^b^ ‘Uni’ indicating univariate analysis while ‘Multi’ indicating multivariate analysis

^c^N/A indicating not applicable due to only one study available

## Discussion

This systematic review demonstrated that RA and PM/DM were the two major underlying diseases that developed AE of systemic autoimmune disease-ILD. The frequency of AE of systemic autoimmune disease-ILD was diverse between studies, which was distributed between 4.3 and 32.9% and the incident rate was 3.19 and 5.77 per 100 patient-years in two studies. The 90-day all-cause mortality of AE of systemic autoimmune disease-ILD was between 30.0 and 58.3%. Older age at initial presentation and lower %DLCO were associated with the development of AE of systemic autoimmune disease-ILD while lower PaO_2_/FiO_2_ at the onset of AE was related to all-cause mortality of the disease.

AE of IP was first reported as small case series where acute worsening of IPF beyond its usually anticipated gradual progression was presented [11]. Subsequently, it was noted that this phenomenon is not unique to IPF but also develops in other fibrotic IPs including systemic autoimmune disease-ILD [12]. The incidence of AE of IPF was estimated in a number of previous reports. Based on data of placebo arms of randomised controlled trials, it was 15.7 per 100 patient-years in one study [66] and 7.6% over 52 weeks in another study [67]. In cohort studies the incidence of AE of IPF was reported to be 8.5% and 14.2% at 1 year [68, 69]. Conversely, the incidence of AE of systemic autoimmune disease-ILD was estimated as 3.19 and 5.77 per 100 patient-years in two studies in this review [45, 55]. Although other two studies also reported the frequency of AE of systemic autoimmune disease-ILD as 7.2% [62] and 21.6% [51], their follow-up periods were as long as 6.0 and 8.5 years, respectively. Therefore, the incidence of AE of systemic autoimmune disease-ILD seems to be lower than AE of IPF. This difference may be related to the underlying pathological IP patterns. IPF is characterised by UIP pattern whereas NSIP is the most prevalent pattern in the majority of systemic autoimmune disease-ILDs [13]. As AE is complicated more frequently in UIP than NSIP regarding IIPs [70], a lower percentage of UIP pattern for systemic autoimmune disease-ILD may have led to a lower number of AE cases for the disease. In fact, a radiological UIP pattern was selected as a potential risk factor and found to be positively associated with the development of AE of systemic autoimmune disease-ILD by a meta-analysis and univariate results of other non-pooled studies although it was not confirmed by a multivariate analysis. A recent systematic review reported that FVC was considered as a risk factor of AE of IPF whereas DLCO might be unrelated to this condition [71]. Conversely, in the current study, DLCO was deemed as a risk factor of AE of systemic autoimmune disease-ILD whereas FVC was not predictive of the disease. DLCO is a sensitive marker for IPs and reflects abnormalities of pulmonary parenchyma [72]. It is also affected by pulmonary hypertension (PH) [73], which is reported to be associated with AE of IPF [74]. Although PH was not selected as a potential risk factor of AE of systemic autoimmune disease-ILD in this review due to a small number of reports, it often complicates the disease and may possibly have confounded the effect of DLCO or vice versa. In contrast, FVC is a reliable index for a progression of IPF [75]. As fibrotic changes progress, the affected lungs become more vulnerable to external stimuli such as respiratory infection and gastric aspiration, which are noted to be a trigger of AE [76, 77]. A previous study reported that FVC was higher and the extent of honeycomb was smaller for patients with AE of systemic autoimmune disease-ILD compared to those with AE of IPF [50]. Therefore, AE may suddenly occur even in the early stage of systemic autoimmune disease-ILD whereas AE of IPF may develop more often in advanced stage, which may cause a dissociation of FVC and DLCO between AE of systemic autoimmune disease-ILD and that of IPF [50]. A prior treatment with corticosteroid or immunosuppressive agents was not deemed as a risk factor of AE of systemic autoimmune disease-ILD in this review. A long-standing immunosuppressive state under these treatments would predispose patients to respiratory infection, which can cause the development of AE. Conversely, an early treatment of systemic autoimmune disease-ILD may be beneficial to delay a progression of the disease and thus protect the lung from developing AE. Although some therapeutic agents, in particular, methotrexate is reported to worsen ILD [51], its usage was not selected as a potential risk factor in this review due to a small number of reports. Similarly, there was scarce literature regarding the effect of biological and anti-fibrotic agents as well as the extent of ILD regarding the development of AE of systemic autoimmune disease-ILD. However, anti-fibrotic agents, in particular, nintedanib is reported to reduce the frequency of AE of IPF [78] and this therapeutic agent is now available to treat progressive fibrosing-ILD including systemic autoimmune disease-ILD [79]. Therefore, this class of medicine seems to be promising to reduce the incidence of AE of systemic autoimmune disease-ILD. The clinical significance of anti-fibrotic agents needs to be elucidated in the future study.

The 90-day all-cause mortality of AE of systemic autoimmune disease-ILD was between 30.0 and 58.3% in this study, which was similar to that of AE of IPF [80]. This finding was consistent with a previous study that reported no difference in the mortality among AE of IPs regardless of whether IPF or other fibrosing IPs [45]. The pathogenesis of AE of IP has yet to be fully understood and it remains unanswered if there is any prognostic difference between AE of IPF and AE of systemic autoimmune disease-ILD. However, both diseases are characterised by the same pathological change, i.e., diffuse alveolar damage, which is a destruction of alveolar epithelium accompanied by a loss of integrity of endothelial cells, causing disordered coagulopathy and fibrinolysis and fibrin deposition [81]. These pathological abnormalities caused by AE are the common features of this unique condition regardless of underlying diseases and can explain poor prognosis of AE of systemic autoimmune disease-ILD. Another possible explanation of poor prognosis of AE of systemic autoimmune disease-ILD may be a delay of initiating a proper treatment for those who previously received pharmacological therapy using corticosteroid or immunosuppressive agents because infections or drug-induced lung diseases often need to be differentiated under these circumstances, which may be a complicated process that takes some time. PaO_2_/FiO_2_ at the onset of AE was identified as a prognostic factor of AE of systemic autoimmune disease-ILD in this review, which was consistent with a recent systematic review of AE of IPF [82]. As PaO_2_/FiO_2_ reflects the severity of AE and represents pulmonary state at the onset of the disease, it makes sense that the index can portend the prognosis of AE of systemic autoimmune disease-ILD. Although WBC count at the onset of AE is reported to be predictive of prognosis of AE of IPF [82], it was not selected as a potential prognostic factor of AE of systemic autoimmune disease-ILD in this review due to a small number of reports.

There are some methodological limitations in this study. First, as the most prevalent underlying IP patterns of PM/DM is reported to be NSIP [83] in contrast to the equivalence of RA being UIP [84], the frequency of AE was assumed to be much lower in the former disease than the latter disease. However, RA and PM/DM (including CADM) were identified as the two major underlying diseases that developed AE among systemic autoimmune disease-ILDs. This unexpected finding may be related to misclassification of this condition because DM, in particular, CADM is noted to be often complicated with rapidly progressive IP [85], which may possibly have been confused with AE of underlying chronic fibrotic changes. Another possible explanation may be publication bias or unbalanced previous reports. AE of PM/DM-associated ILD was reported in 9 studies in contrast to AE of RA-associated ILD in 18 studies in this review. However, the largest study of PM/DM consisted of 483 subjects where 64 cases of AE were confirmed [54] whereas that of RA comprised 96 subjects [64]. Therefore, a different sample size between studies may have contributed to the whole number of AE cases. Second, risk and prognostic factors were determined based on the result of multivariate analysis because univariate results in observational studies are usually affected by other confounders [86]. However, only two potential risk factors and one potential prognostic factor were reported using multivariate analysis in three studies and one study, respectively in this review, which were all statistically significant. This means that only a small number of studies impacted the determination of risk and prognostic factors. This may have been caused by a small sample size that prevented multivariate analysis or selective reporting of the result. Third, the generalisability of the findings of this study may be restricted because the majority of studies in this review were conducted in Japan and thus most of the results were based on data of Japanese patients. As it is recognised that Japanese people are more susceptible to some type of IPs [87], their incidence and prognosis of AE of systemic autoimmune disease-ILD may be different from other ethnicities. Finally, all studies in this review were subject to some shortcomings, causing study limitations that lowered the level of evidence generated in this review to low or very low. Therefore, a high-quality study with a larger sample size is imperative to overcome all of these limitations. A larger number of research focusing on a wide range of systemic autoimmune disease-ILDs also needs to be conducted in a region outside Asia such as North America and Europe to confirm the findings of this study.

## Conclusions

AE of systemic autoimmune disease-ILD was not uncommon and the prognosis of the disease was dismal. Older age at initial presentation and lower %DLCO were deemed predictive of AE of systemic autoimmune disease-ILD while PaO_2_/FiO_2_ at the onset of AE was considered as a prognostic factor of the disease.

## Supplementary Information

**Additional file 1**. Search terms for each electronic database

**Additional file 2**. Clinical information that was not selected as potential risk or prognostic factors

**Additional file 3**. Preferred Reporting Items for Systematic Review and Meta-Analysis

**Additional file 4**. Meta-analysis of Observational Studies in Epidemiology statement

## Data Availability

The dataset used and/or analysed during the current study will be available from the corresponding author on a reasonable request after the final result is published in a journal.

## Abbreviations

AE: Acute exacerbation
ANCA: Anti-neutrophil cytoplasmic antibody
CADM: Clinically amyopathic dermatomyositis
CDSR: Cochrane database of systematic reviews
CI: Confidence interval
CRP: C-reactive protein
DLCO: Diffusing capacity of the lung for carbon monoxide
FVC: Forced vital capacity
GGO: Ground glass opacity
GRADE: Grades of recommendation assessment development and evaluation
HR: Hazard ratio
HRCT: High resolution computed tomography
IIP: Idiopathic interstitial pneumonia
ILD: Interstitial lung disease
IP: Interstitial pneumonia
IPAF: Interstitial pneumonia with autoimmune features
IPF: Idiopathic pulmonary fibrosis
KL-6: Krebs von den Lungen-6
LDH: Lactate dehydrogenase
MCTD: Mixed connective tissue disease
MD: Mean difference
MOOSE: Meta-analysis of observational studies in epidemiology
MPA: Microscopic polyangiitis
NSIP: Non-specific interstitial pneumonia
OR: Odds ratio
PaO_2_/FiO_2_: Partial pressure of arterial oxygen to fraction of inspired oxygen
PI: Prediction interval
PM/DM: Polymyositis/dermatomyositis
PRISMA: Preferred reporting items for systematic review and meta-analysis
QUIPS: Quality in prognostic studies
RA: Rheumatoid arthritis
RevMan: Review manager
RR: Risk ratio
SS: Sjögren’s syndrome
SLE: Systemic lupus erythematosus
SSc: Systemic sclerosis
UIP: Usual interstitial pneumonia
