# Supplementary material for: A systematic review of the incidence, risk factors and prognosis of acute exacerbation of systemic autoimmune disease-associated interstitial lung disease
Source: BMC Pulm Med. 2021 May 5;21:150. doi: 10.1186/s12890-021-01502-w (PMC8101129; doi:10.1186/s12890-021-01502-w)
Supplement: Supplementary file 1 — Additional file 1. Search terms for each electronic database [file 12890_2021_1502_MOESM1_ESM.docx]

**Title**

A systematic review of the incidence, risk factors and prognosis of acute exacerbation of connective tissue disease-associated interstitial pneumonia

**Authors**

Hiroyuki Kamiya^1*^, Ogee Mer Panlaqui^2^

^1^Department of Respiratory Medicine, Tatebayashi Kosei Hospital, Gunma, Japan

^2^Department of Intensive Care Medicine, Northern Hospital, Melbourne[, Australia](https://www.bing.com/local?lid=YN3724x6466142562232593215&id=YN3724x6466142562232593215&q=Epworth+Richmond&name=Epworth+Richmond&cp=-37.8172454833984%7e144.993225097656&ppois=-37.8172454833984_144.993225097656_Epworth+Richmond&FORM=SNAPST)

*Correspondence

Hiroyuki Kamiya

Department of Respiratory Medicine, Tatebayashi Kosei Hospital

262-1 Narushima-cho, Tatebayashi, Gunma, Japan 374-8533

Phone: +81-276-72-3140

Email: mlb04194@nifty.com

e-Appendix: Search terms for each electronic database

Medline (Ovid)

1 exp Lung Diseases, Interstitial/

2 exp Pulmonary Fibrosis/

3 (interstitial adj3 lung adj3 disease?).mp.

4 (pulmonary adj3 fibros$).mp.

5 (interstitial adj3 pneumoni$).mp.

6 alveolitis.mp.

7 exp Connective Tissue Diseases /

8 (connective tissue disease?).mp.

9 (connective tissue disorder).mp.

10 exp Rheumatic Diseases /

11 (rheumat$ disease?).mp.

12 exp Arthritis, Rheumatoid /

13 (rheumatoid arthritis).mp.

14 exp Scleroderma, Systemic /

15 (scleroderma).mp.

16 (systemic sclerosis).mp.

17 exp Polymyositis /

18 exp Dermatomyositis /

19 (polymyositis).mp.

20 (dermatomyositis).mp.

21 exp Lupus Erythematosus, Systemic /

22 (systemic adj3 lupus erythematosus).mp.

23 exp Mixed Connective Tissue disease /

24 (mixed connective tissue disease).mp.

25 (mixed collagen disease).mp.

26 (overlap syndrome).mp.

27 exp Sjogren’s Syndrome /

28 (sjo?gren$ syndrome).mp.

29 exp Disease Progression /

30 (acute adj3 exacerbation?).mp.

31 (disease adj3 progression?).mp.

32 (disease adj3 exacerbation?).mp.

33 (deterioration?).mp.

34 (1 or 2 or 3 or 4 or 5 or 6)

35 (7 or 8 or 9 or 10 or 11 or 12 or 13 or 14 or 15 or 16 or 17 or 18 or 19 or 20 or 21 or 22 or 23 or 24 or 25 or 26 or 27 or 28)

36 (29 or 30 or 31 or 32 or 33)

37 (34 and 35 and 36)

38limit 37 to yr="2002 -Current"

EMBASE (Ovid)

1 exp interstitial lung disease/

2 exp interstitial pneumonia/

3 exp fibrosing alveolitis/

4 exp lung fibrosis /

5 (interstitial adj3 lung adj3 disease?).mp.

6 (interstitial adj3 pneumoni$).mp.

7 (pulmonary adj3 fibros$).mp.

8 alveolitis.mp.

9 exp connective tissue disease /

10 (connective tissue disease?).mp.

11 (connective tissue disorder).mp.

12 exp collagen disease /

13 exp rheumatic disease /

14 (rheumat$ disease?).mp.

15 exp rheumatoid arthritis /

16 (rheumatoid arthritis).mp.

17 exp scleroderma /

18 exp diffuse scleroderma /

19 exp systemic sclerosis /

20 (scleroderma).mp.

21 (systemic sclerosis).mp.

22 exp polymyositis /

23 exp dermatomyositis /

24 (polymyositis).mp.

25 (dermatomyositis).mp.

26 exp systemic lupus erythematosus /

27 (systemic adj3 lupus erythematosus).mp.

28 exp mixed connective tissue disease /

29 (mixed connective tissue disease).mp.

30 (mixed collagen disease).mp.

31 (overlap syndrome).mp.

32 exp Sjoegren syndrome /

33 (sjo?gren$ syndrome).mp.

34 exp disease exacerbation /

35 exp deterioration /

36 (acute adj3 exacerbation?).mp.

37 (disease adj3 progression?).mp.

38 (disease adj3 exacerbation?).mp.

39 (deterioration?).mp.

40 (1 or 2 or 3 or 4 or 5 or 6 or 7 or 8)

41 (9 or 10 or 11 or 12 or 13 or 14 or 15 or 16 or 17 or 18 or 19 or 20 or 21 or 22 or 23 or 24 or 25 or 26 or 27 or 28 or 29 or 30 or 31 or 32 or 33)

42 (34 or 35 or 36 or 37 or 38 or 39)

43 (40 and 41 and 42)

44 limit 43 to yr="2002 -Current"

Science Citation Index Expanded (Web of Science Core Collection)

#1 TS=("interstitial NEAR/3 lung NEAR/3 disease$") OR TS=("interstitial NEAR/3 pneumonia$") OR TS=(alveolitis) OR TS=("pulmonary NEAR/3 fibros*")

#2 TS=("connective tissue dis*") OR TS=("rheumat* disease") OR TS=("rheumatoid arthritis") OR TS=("scleroderma") OR TS=("systemic sclerosis") OR TS=("polymyositis") OR TS=("dermatomyositis") OR TS=("systemic NEAR/3 lupus erythematosus") OR TS=("mixed connective tissue dis*") OR TS=("overlap syndrome") OR TS=("sj*gren* syndrome")

#3 TS=(acute NEAR/3 exacerbation$) OR TS=(disease NEAR/3 progression$) OR TS=(disease NEAR/3 exacerbation$) OR TS=(deterioration$)

#4 #1 AND #2 AND #3

#5 #4 AND (2002-2020)

Google scholar

(“interstitial lung disease” OR “interstitial pneumonia” OR “pulmonary fibrosis”) (“connective tissue disease” OR “connective tissue disorder” OR “rheumatic disease”) (“acute exacerbation” OR "disease progression" OR "disease exacerbation")
