# Supplementary material for: A systematic review of the incidence, risk factors and prognosis of acute exacerbation of systemic autoimmune disease-associated interstitial lung disease
Source: BMC Pulm Med. 2021 May 5;21:150. doi: 10.1186/s12890-021-01502-w (PMC8101129; doi:10.1186/s12890-021-01502-w)
Supplement: Supplementary file 2 — Additional file 2. Clinical information that was not selected as potential risk or prognostic factors [file 12890_2021_1502_MOESM2_ESM.docx]

e-Table Clinical information that was not selected as potential risk or prognostic factors^a^

|  | Incidence | | | | | Prognosis |
| --- | --- | --- | --- | --- | --- | --- |
| Study | Pulmonary hypertension | Extent of ILD | Biologics | Anti-fibrotics | ILD preceding | WBC count (at AE) |
| Isobe 2010 [41] | - | - | - | - | - | - |
| Lim 2019 [42] | - | - | - | - | - | - |
| Park 2007 [43] | - | - | - | - | - | Median 13000 (range 5900-17100)/µL (patients with AE) |
| Song 2012 [44] | - | MD 0.39 (-0.75-1.52) (Fibrosis score)^b^ | - | - | 7/84 patients (all) | - |
| Suzuki 2019 [45] | - | - | - | - | - | - |
| Tachikawa 2011 [46] | - | - | - | - | - | - |
| Tomiyama 2016 [47] | 2/13 patients with AE | - | - | - | - | - |
| Akiyama 2016 [48] | - | - | - | - | - | - |
| Cao 2019 [49] | HR 1.03 (0.99-1.07) | - | - | - | - | HR 1.10 (1.03-1.18) |
| Enomoto 2018 [50] | - | - | - | - | - | - |
| Hozumi 2013 [51] | - | - | - | - | - | - |
| Ichiyasu 2017 [52] | - | - | - | - | - | - |
| Ishikawa 2017 [53] | - | - | - | - | - | - |
| Liang 2020 [54] | - | - | - | - | - | - |
| Manfredi 2019 [55] | - | - | - | - | 29/78 patients (all) | - |
| Okamoto 2016 [56] | - | - | - | - | - | - |
| Ota 2017 [57] | - | - | - | - | 6/12 patients with AE | - |
| Parambil 2006 [58] | - | - | - | - | - | - |
| Silva 2007 [59] | - | - | - | - | - | - |
| Singh 2019 [60] | - | - | - | - | - | - |
| Su 2017 [61] | - | 15.9±7.6 (mean±SD) (all patients) (HRCT score)^c^ | - | - |  | - |
| Suda 2009 [62] | - | - | - | - |  | - |
| Toyoda 2016 [63] | - | - | - | - |  | - |
| Yamakawa 2019[64] | - | - | 19/96 patients (all) | 3/96 patients (all) | 16/96 patients (all) | - |

Relative values express the risk associated with the presence of a certain factor and other figures indicate the number of positivity among all patients with interstitial lung disease (ILD) or patients with acute exacerbation of ILD unless otherwise specified.

a. clinical information that may be relevant to the incidence or prognosis of acute exacerbation of systemic autoimmune disease-associated ILD but was not selected as a risk or prognostic factor due to a small number of reports

b. sum of reticulation and honeycomb score

c. sum of severity and extent score

AE, acute exacerbation; HR, hazard ratio; HRCT, high resolution computed tomography; ILD, interstitial lung disease; MD, mean difference; SD, standard deviation; WBC, white blood cell;
