# Supplementary material for: A systematic review of the incidence, risk factors and prognosis of acute exacerbation of systemic autoimmune disease-associated interstitial lung disease
Source: BMC Pulm Med. 2021 May 5;21:150. doi: 10.1186/s12890-021-01502-w (PMC8101129; doi:10.1186/s12890-021-01502-w)
Supplement: Supplementary file 3 — Additional file 3. Preferred Reporting Items for Systematic Review and Meta-Analysis [file 12890_2021_1502_MOESM3_ESM.doc]

| Checklist items for Meta-analyses of Observational Studies in Epidemiology (MOOSE) | Reported  on Page |
| --- | --- |
| Reporting of background should include |  |
| - Problem definition | Page 6-7 |
| - Hypothesis statement | Not described |
| - Description of study outcome(s) | Page 10-11 |
| - Type of exposure or intervention used | Page 10 |
| - Type of study designs used | Page 11 |
| - Study population | Page 8-10 |
| Reporting of search strategy should include |  |
| - Qualifications of searchers (eg, librarians and investigators) | Page 11 |
| - Search strategy, including time period included in the synthesis and keywords | Page 11-12  e-Appendix |
| - Effort to include all available studies, including contact with authors | Page 12 |
| - Databases and registries searched | Page 11-12 |
| - Search software used, name and version, including special features used (eg, explosion) | Not described |
| - Use of hand searching (eg, reference lists of obtained articles) | Page 12 |
| - List of citations located and those excluded, including justification | Figure 1 |
| - Method of addressing articles published in languages other than English | Page 11 |
| - Method of handling abstracts and unpublished studies | Page 11 |
| - Description of any contact with authors | Not described |
| Reporting of methods should include |  |
| - Description of relevance or appropriateness of studies assembled for assessing the hypothesis to be tested | Not described |
| - Rationale for the selection and coding of data (eg, sound clinical principles or convenience) | Not described |
| - Documentation of how data were classified and coded (eg, multiple raters, blinding, and interrater reliability) | Not described |
| - Assessment of confounding (eg, comparability of cases and controls in studies where appropriate) | Not described |
| - Assessment of study quality, including blinding of quality assessors; stratification or regression on possible predictors of study results | Page 13 |
| - Assessment of heterogeneity | Page 15-16 |
| - Description of statistical methods (eg, complete description of fixed or random effects models, justification of whether the chosen models account for predictors of study results, dose-response models, or cumulative meta-analysis) in sufficient detail to be replicated | Page 14-15 |
| - Provision of appropriate tables and graphics | Figure 1 (study flow diagram) |
| Reporting of results should include |  |
| - Graphic summarizing individual study estimates and overall estimate | Table 1,3,4,5 |
| - Table giving descriptive information for each study included | Table 1 |
| - Results of sensitivity testing (eg, subgroup analysis) | Page 22 |
| - Indication of statistical uncertainty of findings | Page 20-22  Table 3,4,5 |
| Reporting of discussion should include |  |
| - Quantitative assessment of bias (eg, publication bias) | Not described |
| - Justification of exclusion (eg, exclusion of non-English-language citations) | Not described |
| - Assessment of quality of included studies | Page 22 |
| Reporting of conclusions should include |  |
| - Consideration of alternative explanations for observed results | Page 23-27 |
| - Generalization of the conclusions (ie, appropriate for the data presented and within the domain of the literature review) | Page 29 |
| - Guidelines for future research | Page 29 |
| - Disclosure of funding source | Page 32 |

From Stroup DF, Berlin JA, Morton SC, et al. Meta-analysis of observational studies in epidemiology: a proposal for reporting. Meta-analysis Of Observational Studies in Epidemiology (MOOSE) group. JAMA 2000;283:2008-12.

**Title**

A systematic review of the incidence, risk factors and prognosis of acute exacerbation of systemic autoimmune disease-associated interstitial pneumonia

**Authors**

Hiroyuki Kamiya^1*^, Ogee Mer Panlaqui^2^

^1^Department of Respiratory Medicine, Tatebayashi Kosei Hospital, Gunma, Japan

^2^Department of Intensive Care Medicine, Northern Hospital, Melbourne[, Australia](https://www.bing.com/local?lid=YN3724x6466142562232593215&id=YN3724x6466142562232593215&q=Epworth+Richmond&name=Epworth+Richmond&cp=-37.8172454833984~144.993225097656&ppois=-37.8172454833984_144.993225097656_Epworth+Richmond&FORM=SNAPST)

*Correspondence

Hiroyuki Kamiya

Department of Respiratory Medicine, Tatebayashi Kosei Hospital

262-1 Narushima-cho, Tatebayashi, Gunma, Japan 374-8533

Phone: +81-276-72-3140

Email: mlb04194@nifty.com
